# Validation of pathological grading systems for predicting metastatic potential in pheochromocytoma and paraganglioma

**DOI:** 10.1371/journal.pone.0187398

**Published:** 2017-11-08

**Authors:** Jung-Min Koh, Seong Hee Ahn, Hyeonmok Kim, Beom-Jun Kim, Tae-Yon Sung, Young Hoon Kim, Suck Joon Hong, Dong Eun Song, Seung Hun Lee

**Affiliations:** 1 Division of Endocrinology and Metabolism, Asan Medical Center, University of Ulsan College of Medicine, Seoul, Korea; 2 Department of Endocrinology, Inha University School of Medicine, Incheon, Korea; 3 Division of Endocrinology and Metabolism, Department of Medicine, Gyeongsang National University School of Medicine, Jinju, Korea; 4 Department of Surgery, Asan Medical Center, University of Ulsan College of Medicine, Seoul, Korea; 5 Department of Pathology, Asan Medical Center, University of Ulsan College of Medicine, Seoul, Korea; 2nd medical school of Charles University, CZECH REPUBLIC

## Abstract

**Purpose:**

The Grading system for Adrenal Pheochromocytoma and Paraganglioma (GAPP) was proposed for predicting the metastatic potential of pheochromocytoma and paraganglioma to overcome the limitations of the Pheochromocytoma of the Adrenal Scaled Score (PASS). However, to date, no study validating the GAPP has been conducted, and previous studies did not include mutations in the succinate dehydrogenase type B (SDHB) gene in the score calculation. In this retrospective cohort study, we validated the prediction ability of GAPP and assessed whether it would be improved by inclusion of the loss of SDHB immunohistochemical staining.

**Methods:**

We divided the tumors into non-metastatic and metastatic groups based on the presence of synchronous or metachronous metastases. The GAPP score and PASS at the initial operation were measured. Moreover, we combined some GAPP parameters with the immunohistochemical staining of SDHB to obtain a modified GAPP (M-GAPP) score.

**Results:**

Metastasis occurred in 15/72 (20.8%) patients, with a mean follow-up of 43.5 months. Loss of SDHB staining was more frequent (*P* = 0.044) in the metastatic group. The GAPP score (*P* = 0.006), PASS (*P* = 0.003), and M-GAPP score (*P*<0.001) were all higher in the metastatic group. Twelve of 40 (30.0%) moderately or poorly differentiated tumors, as defined by the GAPP score, and 12/34 (35.3%) tumors with a PASS ≥4 were metastatic. Conversely, 10/19 (52.6%) tumors with an M-GAPP score ≥3 were metastatic. The area under the curve of the M-GAPP score (0.822) was significantly higher than that of the GAPP (0.728) (*P* = 0.012), but similar to that of the PASS (0.753) (*P* = 0.411). The GAPP (*P* = 0.032) and M-GAPP scores (*P* = 0.040), but not PASS (*P* = 0.200), negatively correlated with metastasis-free survival.

**Conclusion:**

The GAPP was validated, and M-GAPP, a combination of some GAPP parameters and loss of SDHB staining, might be useful for the prediction of the metastatic potential of pheochromocytoma and paraganglioma.

## Introduction

Pheochromocytoma and paragangliomas (PPGLs) are rare catecholamine-secreting neuroendocrine tumors that arise from chromaffin cells of the adrenal medulla and extra-adrenal sites, respectively [1]. The majority of PPGLs are benign; however, approximately 10% of pheochromocytomas (PHEO) and 15–35% of paragangliomas (PGL) are malignant [2]. The current definition of malignancy by the World Health Organization is only the presence of metastases in non-chromaffin tissue [3, 4]. The 5-year overall survival rate of patients with metastases ranges from 30–92% [2, 5, 6], whereas that of patients without metastases is 89.3% [7]. The main cause of death during the follow-up period is metastasis occurrence, even in patients who were initially diagnosed with benign PPGLs [7]. Therefore, it is very important to predict the metastatic potential of PPGLs, because such cases should be followed-up more aggressively.

Attempts to develop effective systems for predicting the metastatic potential of PPGLs using multiple histological parameters have been made, because no individual findings have been shown to be sufficiently reliable to allow a tumor to be confidently dismissed as benign. For example, the Pheochromocytoma of the Adrenal Scaled Score (PASS) [8] and the Grading system for Adrenal Pheochromocytoma and Paraganglioma (GAPP) [9] have been used in previous studies. The PASS is a weighted score comprising 12 specific histological features that are more frequently identified in metastatic pheochromocytoma [8]. PASS has some limitations such as its application to only pheochromocytoma and high inter-observer and intra-observer variations, even by expert pathologists [10]. To overcome this, the GAPP was recently developed [9]. This score excludes some of the poorly concordant histological features in the PASS and additionally includes the biochemical phenotype [11]. However, to date, there has been no validation study of the GAPP system [11, 12], and its clinical use is thus limited. More importantly, the lack of the inclusion of mutations in the succinate dehydrogenase gene subunit B (SDHB) was pointed out as a limitation of the GAPP system [11, 12], because SDHB mutations are well known to be strongly correlated with the metastatic potential of PPGL [1]. Several studies have shown that SDHB gene mutations can be detected by the loss of SDHB staining on immunohistochemistry (IHC) [13, 14]. Although one study did not include this factor in the GAPP grading system, it suggested that a combination of loss of SDHB staining with GAPP might be useful to predict metastatic potential [9]. Therefore, in this retrospective cohort study, we aimed to (1) validate the GAPP and (2) determine the improvement in predictive ability by using a modified GAPP (M-GAPP) score, comprising a combination of the loss of SDHB staining with some GAPP parameters, by comparing it with the PASS and GAPP score.

## Materials and methods

### Patients and tissues

The study population consisted of 72 PPGL patients who were diagnosed at Asan Medical Center, Seoul, Korea from July 2007 to August 2016 (S1 File). Paragangliomas of the head and neck usually arise from parasympathetic neuronal tissue but have different behaviors, such as a lack of secretion of catecholamines, and were therefore excluded from this study. We obtained the patients’ clinical information, including sex, age at initial diagnosis, location of primary tumor, and 24-hour urinary metanephrines secretion.

All study participants provided written informed consent. This study was approved by the Institutional Review Board of Asan Medical Center.

### Biochemical testing

We measured urine metanephrines, including 24-hour urinary fractionated metanephrine (UMN) and normetanephrine (UNM), using the Agilent 1100 high-performance liquid chromatography system (Agilent Technologies, Santa Clara, CA, USA) with an electrochemical detector, and using a high-performance liquid chromatography kit (Chromsystems, Munich, Germany). A tumor was defined as functional when the UMN or UNM was elevated. We used symptom-dependent cut-offs of urine metanephrines, as previously described [15].

### Baseline histological analysis

For the histological analysis, tumor size was grossly measured as the maximum diameter of the tumor specimen. All hematoxylin and eosin-stained slides of the 72 surgically resected PPGL specimens were reviewed by an experienced endocrine pathologist based upon the GAPP scoring system classification (Table 1) [9] and PASS (S1 Table) [8] in a blinded manner without knowledge of the clinical outcome. The tumors were classified into 3 differentiation types according to their GAPP scores: well differentiated (WD; 0–2), moderately differentiated (MD; 3–6) and poorly differentiated (PD; 7–10). A PASS ≥4 was defined as having increased metastatic potential, as compared to PASS <4.

**Table 1:** Grading system for Adrenal Pheochromocytoma and Paraganglioma (GAPP).

GAPP parameters	Points scored
Histological pattern	
Zellballen	0
Large and irregular cell nest	1
Pseudorosette (even focal)	1
Comedo-type necrosis	
Absence	0
Presence	2
Cellularity	
Low (<150 cells/U)	0
Moderate (150–250 cells/U)	1
High (>250 cells/U)	2
Ki67 labeling index (%)	
<1	0
1–3	1
>3	2
Vascular or capsular invasion	
Absence	0
Presence	1
Catecholamine type	
Non-functioning	0
Adrenergic type	0
Noradrenergic type	1
Total maximum score	10

If the urine fractionated metanephrine (UMN) levels were high with or without elevated urine fractionated normetanephrine (UNM) levels, the catecholamine type was adrenergic type.

If the UNM levels were high without elevated UMN levels, the catecholamine type was noradrenergic type.

IHC staining for Ki-67 was performed using an automated IHC staining instrument (Benchmark; Ventana Medical Systems, Tucson, USA), the UltraView Universal DAB kit (Ventana Medical Systems), and a Ki-67 antibody (clone MIB-1, 1:200 dilution; DAKO, Glostrup, Denmark). The Ki-67 labeling index was evaluated by a formal manual count to count PPGL cells only. Before counting, the areas for analysis were assessed to select the hottest spot with positively stained PPGL cells, and 2–3 static images were obtained for each case. For all cases, at least 1000 cells were independently and manually evaluated. The number of Ki-67-positive cells per 100 PPGL cells was designated as the labeling index in the hottest spot.

**Fig 1:**
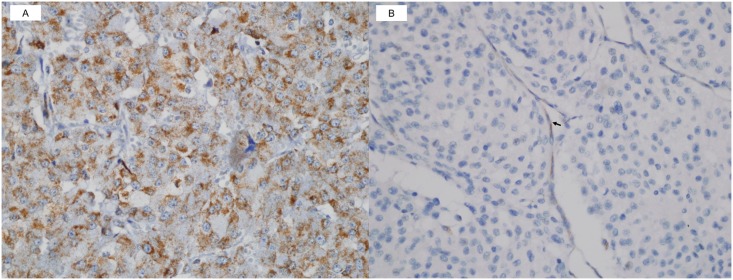
Immunohistochemical (IHC) staining of succinate dehydrogenase gene subunit B (SDHB). (A) Positive SDHB IHC staining with definite granular cytoplasmic staining (mitochondrial pattern) and (B) negative SDHB IHC staining without definite granular cytoplasmic staining, in contrast to positive staining in the internal controls such as endothelial cells (arrow) and sustentacular cells (SDHB IHC original magnification, ×400).

IHC staining of SDHB was also performed using an automated IHC staining instrument (Benchmark; Ventana Medical Systems), the UltraView Universal DAB kit (Ventana Medical Systems), and an SDHB antibody (rabbit polyclonal HPA002868, 1:400 dilution; Sigma—Aldrich, St Louis, MO, USA). Cases with any definite granular cytoplasmic staining (mitochondrial pattern) were scored as positive (Fig 1A). The proportion of positive granular cytoplasmic staining varied greatly between positive cases. Weak diffuse cytoplasmic staining was occasionally and heterogeneously observed in combination with definite granular cytoplasmic staining, which was scored as ‘positive’. Cases with completely absent staining or only weak diffuse cytoplasmic staining, in contrast to the positive internal controls (endothelial cells, sustentacular cells, and lymphocytes), were scored as ‘negative’ (Fig 1B).

### Definition and clinical assessment of metastatic PPGL

Metastatic PPGL was defined as the presence or recurrence of metastatic lesions at sites where neuroendocrine tissue is normally absent [4]. For intra-abdominal metastasis, metastatic PPGL was defined as only the occurrence of lymph node metastasis to rule out pheochrocytomatosis [16]. Most metastatic lesions were confirmed by histologic evidence except for 5 patients who were confirmed by computed tomography and ^123^I-meta-iodo-benzyl-guanidine imaging, as well as by functional studies, as metastatic PPGLs. Patients with metastases were subdivided into two groups, synchronous and metachronous metastases, defined as those who had metastatic lesions at the time of or <6 months after diagnosis of the primary tumor and those who developed metastases ≥ 6 months after the initial time of diagnosis and/or resection of the primary tumor, respectively [3].

### Statistical analysis

Data are presented as the mean±standard deviation, median [interquartile range (IQR)], or as numbers (percentages). The patients’ baseline characteristics were compared using Student’s *t* test or the Mann-Whitney U-test for continuous variables, or the chi-square test for categorical variables. Univariate and multivariate Cox proportional hazards regression models were evaluated to assess the association of each parameter of the GAPP, PASS, and M-GAPP with the risk of malignancy. The abilities of the GAPP score, PASS, and M-GAPP score to predict malignancy were quantified using the area under the curve (AUC) from receiver-operating characteristic (ROC) analysis. The metastasis-free survival (MFS) was defined as the interval between surgery and the date of diagnosis of the first metastasis. Correlations of the GAPP score, PASS, and M-GAPP score with the MFS were analyzed using Pearson’s correlation analyses. We used the Kaplan-Meier method to estimate the MFS and the log-rank test to compare the MFS between the groups. All tests were 2-sided, and *P*<0.05 was considered statistically significant. All statistical analyses were performed using SPSS version 18.0 (SPSS Inc., Chicago, IL, USA).

## Results

### Characteristics of the 72 PPGL patients

With a mean follow-up duration of 43.5 months after the initial operation, metastases occurred in 15 of 72 (20.8%) patients, including 5 (6.9%) synchronous and 10 (13.9%) metachronous metastases (Table 2). The age tended to be younger (*P* = 0.061) and tumor size tended to be larger (*P* = 0.060) in the metastatic than in the non-metastatic group. Patients with only the adrenergic secretory type tended to be more common than those with the noradrenergic secretory type (*P* = 0.097) in the metastatic than in the non-metastatic group. There were no significant differences in the levels of urine metanephrines between the two groups. A loss of SDHB staining was observed in 11 (15.3%) tumors, and was significantly more frequent in the metastatic (5 of 15, 33.3%) than in the non-metastatic group (6 of 57, 10.5%) (*P* = 0.044). The follow-up duration tended to be longer in the non-metastatic than in the metastatic group (*P* = 0.083).

**Table 2:** Baseline characteristics of the 72 PPGL patients according to the occurrence of metastasis.

Variable	PPGL (*N* = 72)	Non-metastatic PPGL (*N* = 57)	Metastatic PPGL (*N* = 15)	*P*
Age (years), mean±SD	46.0±15.3	47.8±14.5	39.5±16.7	0.061
Female, *N* (%)	57 (79.2%)	47 (82.5%)	10 (66.7%)	0.326
Height (cm), mean±SD	159.0±7.6	158.1±7.7	162.4±6.7	0.052
Weight (kg), mean±SD	59.7±9.4	59.4±9.7	60.9±8.6	0.586
BMI (kg/m^2^), mean±SD	23.6±3.3	23.7±3.4	23.1±3.1	0.496
Size of tumor (cm), mean±SD	7.0±3.5	6.6±3.3	8.5±4.1	0.060
PGL, *N* (%)	9 (12.5%)	7 (12.3%)	2 (13.3%)	0.999
Familial form, *N* (%)	6 (8.3%)	4 (7.0%)	2 (13.3%)	0.793
Functioning type, *N* (%)	60 (83.3%)	47 (82.5%)	13 (86.7%)	>0.999
Adrenergic type, *N* (%)	39 (65.0%)	33 (70.2%)	6 (46.2%)	0.097
Noradrenergic type, *N* (%)	21 (35.0%)	14 (29.8%)	7 (53.8%)	
UMN (μg/day), median [IQR]	1087.7 [142.7; 3002.5]	1013.4 [138.6; 2876.3]	1373.2 [669.7; 3429.4	0.459
UNM (μg/day), median [IQR]	3510.0 [1965.3; 7132.5]	3510.0 [2234.6; 7206.8]	4000.3 [2411.6; 5060.3]	0.732
Loss of SDHB staining on IHC, *N* (%)	11 (15.3%)	**6 (10.5%)**	**5 (33.3%)**	**0.044**
Metastases, *N* (%)	15 (20.8%)			
Synchronous metastases, *N* (%)	5 (6.9%)			
Metachronous metastases, *N* (%)	10 (13.9%)			
Duration of follow-up (months), mean±SD	43.5±37.4	46.8±38.7	30.8±28.7	0.083

BMI, body mass index; IQR, interquartile ranges; IHC, immunohistochemistry; PHEO, pheochromocytoma; PGL, paraganglioma; PPGL, pheochromocytoma and paraganglioma; SD, standard deviation; SDHB, succinate dehydrogenase gene subunit B; UMN, urine fractionated metanephrine; UNM, urine fractionated normetanephrine.

If the UMN levels were high, with or without elevated UNM levels, the catecholamine type was adrenergic type. If the UNM levels were high, without elevated UMN levels, the catecholamine type was noradrenergic type.

Synchronous metastases were defined as metastatic lesions at the time or <6 months after diagnosis of the primary tumor and metachronous metastases were defined as metastatic lesions ≥ 6 months after the initial time of diagnosis and/or resection of the primary tumor.

Significant results (*P*<0.05) are in bold.

We performed a subsequent analysis separately for PHEO and PGL (Table 3). Metastases occurred in 13 of 73 (20.6%) patients with PHEO and 2 of 7 (22.2%) patients with PGL. The age tended to be younger (P = 0.072) in the metastatic PHEO than in the non-metastatic PHEO group. There were no significant differences in the levels of urine metanephrines and frequency of secretory type between the two groups in both PHEO and PGL patients. A loss of SDHB staining tended to be more frequent in the metastatic PHEO (4 of 13, 30.8%) than in the non-metastatic PHEO group (4 of 50, 8.0%) (P = 0.084).

**Table 3:** Baseline characteristics of the 72 PPGL patients according to the occurrence of metastasis by PHEO or PGL.

Variable	Non-metastatic PHEO (*N* = 50, 79.4%)	Metastatic PHEO (*N* = 13, 20.6%)	*P*	Non-metastatic PGL (*N* = 7, 77.8%)	Metastatic PGL (*N* = 2, 22.2%)	*P*
Age (years), mean±SD	48.8 ± 13.3	40.6 ± 17.8	0.072	40.6 ± 21.3	32.0 ± 2.8	0.606
Female, *N* (%)	44 (88.0%)	8 (61.5%)	0.067	3 (42.9%)	2 (100.0%)	0.530
Height (cm), mean±SD	**157.0 ± 6.9**	**162.2 ± 7.2**	**0.020**	165.9 ± 8.7	163.8 ± 0.4	0.746
Weight (kg), mean±SD	58.1 ±8.2	61.2 ± 9.2	0.251	68.0 ± 14.9	58.7 ± 1.8	0.428
BMI (kg/m^2^), mean±SD	23.6 ±3.4	23.3 ± 3.3	0.714	24.4 ± 3.7	21.9 ± 0.6	0.394
Size of tumor (cm), mean±SD	6.7 ± 3.4	8.5 ± 4.2	0.112	6.1 ± 2.6	9.0 ± 4.9	0.282
Familial form, *N* (%)	4 (8.0%)	2 (15.4%)	0.781	0 (0.0%)	0 (0.0%)	>0.999
Functioning type, *N* (%)	41 (82.0%)	13 (100.0%)	0.227	6 (85.7%)	0 (0.0%)	0.537
Adrenergic type, *N* (%)	30 (73.2%)	6 (46.2%)	0.143	3 (50.0%)	0 (0.0%)	>0.999
Noradrenergic type, *N* (%)	11 (26.8%)	7 (53.8%)		3 (50.0%)	0 (0.0%)	
UMN (μg/day), median [IQR]	1099.2 [161.0; 3518.0]	1373.3 [442.3; 4333.0]	0.644	81.7 [78.9; 91.5]	14.8 [NA]	0.740
UNM (μg/day), median [IQR]	3481.5 [1682.6; 7521.4]	4000.3 [1926.5; 5281.1]	0.878	6045.7 [3510.0; 9873.2]	848.0 [NA]	0.229
Loss of SDHB staining on IHC, *N* (%)	4 (8.0%)	4 (30.8%)	0.084	2 (28.6%)	1 (50.0%)	0.200
Duration of follow-up (months), mean±SD	46.9 ± 37.7	32.6 ± 30.2	0.211	54.1 ± 48.2	26.5 ± 36.1	0.484

BMI, body mass index; IQR, interquartile ranges; IHC, immunohistochemistry; PHEO, pheochromocytoma; PGL, paraganglioma; PPGL, pheochromocytoma and paraganglioma; NA, not applicable; SD, standard deviation; SDHB, succinate dehydrogenase gene subunit B; UMN, urine fractionated metanephrine; UNM, urine fractionated normetanephrine.

If the UMN levels were high, with or without elevated UNM levels, the catecholamine type was adrenergic type. If the UNM levels were high, without elevated UMN levels, the catecholamine type was noradrenergic type.

Significant results (*P*<0.05) are in bold.

### Correlation of the individual parameters of the GAPP score, PASS, and M-GAPP score with the occurrence of metastasis

In the univariate analysis, 4 (67%) of 6 GAPP parameters (i.e., histological pattern, comedo-type necrosis, Ki67 labeling index ≥3%, and noradrenergic type), were significantly different between the metastatic and non-metastatic groups (*P*<0.001–0.029) (Table 4). In the multivariate analysis, 2 (33%) parameters, namely the histological pattern and Ki67 labeling index 1–3%, were significantly different between the two groups (*P* = 0.010–0.029). Of 12 PASS parameters, 5 (42%) in the univariate analysis and 4 (33%) in the multivariate analysis were significantly different between the two groups (S2 Table).

**Table 4:** Association of the individual parameters of the GAPP at the initial operation with occurrence of metastasis.

GAPP parameters	Univariate		Multivariate	
	HR (95% CI)	*P*	HR (95% CI)	*P*
Histological pattern				
Zellballen	Ref		Ref	
Large and irregular cell nest or pseudorosette (even focal)	**4.11 (1.16–14.61)**	**0.029**	**7.00 (1.23–39.89)**	**0.029**
Comedo-type necrosis	**64.08 (7.25–566.26)**	**<0.001**	NA	0.891
Cellularity				
Low (<150 cells/U)	Ref		Ref	
Moderate (150–250 cells/U)	1.08 (0.36–3.24)	0.890	1.04 (0.22–4.90)	0.965
High (>250 cells/U)	2.58 (0.52–12.89)	0.248	0.26 (0.02–3.00)	0.281
Ki67 labeling index (%)				
<1	Ref		Ref	
1–3	2.63 (0.67–10.31)	0.167	**10.70 (1.77–64.68)**	**0.010**
>3	**9.25 (2.16–39.52)**	**0.003**	4.22 (0.66–27.07)	0.128
Vascular or capsular invasion	3.01 (0.85–10.69)	0.089	1.18 (0.26–5.30)	0.830
Catecholamine type				
Non-functioning or adrenergic type	Ref		Ref	
Noradrenergic type	**3.89 (1.33–11.39)**	**0.013**	1.44 (0.29–7.23)	0.660

CI, confidence interval; GAPP, Grading system for Adrenal Pheochromocytoma and Paraganglioma; HR, hazard ratio; NA, not applicable; PPGL, pheochromocytoma and paraganglioma.

If the UMN levels were high, with or without elevated UNM levels, the catecholamine type was adrenergic type. If the UNM levels were high, without elevated UMN levels, the catecholamine type was noradrenergic type.

Significant results (*P* < 0.05) are in bold.

Exclusion of loss of SDHB staining from the GAPP parameters has been established as one of the intrinsic problems with the GAPP. A previous study reported that loss of SDHB staining did not occur in WD PPGLs of the GAPP classification [9]; however, 2 of the 29 (6.9%) non-metastatic PPGLs with WD type showed a loss of SDHB staining in the present study (S3 Table). These findings suggest that a loss of SDHB staining *per se* is not sufficient to predict metastasis; hence, it might be appropriate as one of the parameters in the scoring system. Among the GAPP parameters, there was no significant difference in cellularity, Ki67 labeling index between 1–3% or >3%, and capsular or vascular invasion. Therefore, we reconstructed the M-GAPP using a combination of the loss of SDHB staining with some of the GAPP parameters (Table 5). In the univariate analysis, all 6 (100%) M-GAPP parameters, that is, large and irregular cell nest or pseudorosette, comedo-type necrosis, vascular invasion, Ki67 labeling index ≥1%, noradrenergic type, and loss of SDHB staining, were significantly different between the metastatic and non-metastatic groups (*P*<0.001–0.029) (Table 5). In the multivariate analysis, 3 (50%) of these parameters, namely large and irregular cell nest or pseudorosette, comedo-type necrosis, and Ki67 labeling index ≥1%, remained significantly different between the two groups (*P* = 0.001–0.049).

**Table 5:** Association of the individual parameters of the M-GAPP at the initial operation with occurrence of metastasis.

	Univariate		Multivariate	
	HR (95% CI)	*P*	HR (95% CI)	*P*
Histological pattern				
Zellballen	Ref		Ref	
Large and irregular cell nest or pseudorosette (even focal)	**4.11 (1.16–14.61)**	**0.029**	**4.11 (1.00–17.11)**	**0.049**
Comedo-type necrosis	**64.08 (7.25–566.26)**	**<0.001**	**79.40 (6.22–10.14.48)**	**0.001**
Vascular invasion	**3.48 (1.18–10.26)**	**0.024**	2.95 (0.87–9.99)	0.082
Ki67 labeling index (%)				
<1	Ref		Ref	
≥1	**3.94 (1.17–13.19)**	**0.026**	**4.77 (1.03–22.06)**	**0.046**
Catecholamine type				
Non-functioning or adrenergic type	Ref		Ref	
Noradrenergic type	**3.89 (1.33–11.39)**	**0.013**	1.49 (0.33–6.62)	0.603
SDHB IHC negativity	**3.70 (1.26–10.89)**	**0.018**	1.61 (0.41–6.37)	0.495

CI, confidence interval; HR, hazard ratio; IHC, immunohistochemistry; M-GAPP, Modified Grading system for Adrenal Pheochromocytoma and Paraganglioma; PPGL, pheochromocytoma and paraganglioma; SDHB, succinate dehydrogenase gene subunit B.

If the UMN levels were high, with or without elevated UNM levels, the catecholamine type was adrenergic type. If the UNM levels were high, without elevated UMN levels, the catecholamine type was noradrenergic type.

Significant results (*P* < 0.05) are in bold.

### Comparison of the 3 scoring systems for predicting metastatic potential

The median [IQR] of the GAPP score in the metastatic group (4.0 [3.0 to 6.5]) was higher than that in the non-metastatic group (2.0 [2.0 to 3.0]) (*P* = 0.006) (Table 6). In the case of the GAPP score, 29 of 32 (90.6%) WD PPGLs were non-metastatic, while 12 of 40 (30.0%) MD (*N* = 36) and PD (*N* = 4) PPGLs were metastases (*P*<0.001). In contrast, 35 of 38 (92.1%) PPGLs with a PASS <4 were non-metastatic, and 12 of 34 (35.3%) PPGLs with a PASS ≥4 were metastatic (*P* = 0.010). The median [IQR] of the M-GAPP score in the metastatic group (4.0 [2.0 to 5.0]) was higher than that in the non-metastatic group (1.0 [1.0 to 2.0]) (*P*<0.001). We selected 3 as the best cutoff value of the M-GAPP score, which corresponded to Youden’s index [17] in the receiver-operating characteristics curve analysis. Forty-eight of 53 (90.6%) PPGLs with M-GAPP <3 were non-metastatic, while 10 of 19 (52.6%) PPGLs with M-GAPP ≥3 were metastatic (*P*<0.001). This finding indicates that the negative predictive value for occurrence of metastasis is comparable, while the positive predictive value for occurrence of metastasis is the highest by the M-GAPP scoring system compared to the other two scoring systems.

**Table 6:** Comparison of the GAPP score, PASS, and M-GAPP score for predicting metastatic potential in PPGL.

Variable	Non-metastatic (*N* = 57)	Metastatic (*N* = 15)	*P*
GAPP score, median [IQR]	**2.0 [2.0, 3.0]**	**4.0 [3.0, 6.5]**	**0.006**
WD type (*N* = 32): GAPP score 0–2, n (%)	**29 (90.6%)**	**3 (9.4%)**	**<0.001**
MD type (*N* = 36): GAPP score 3–6, n (%)	**28 (77.8%)**	**8 (22.2%)**	
PD type (*N* = 4): GAPP score 7–10, n (%)	**0 (0.0%)**	**4 (100.0%)**	
PASS, median [IQR]	**4.0 [2.0, 6.0]**	**6.0 [5.0, 8.5]**	**0.003**
PASS <4 (*N* = 38), n (%)	**35 (92.1%)**	**3 (7.9%)**	**0.010**
PASS ≥4 (*N* = 34), n (%)	**22 (64.7%)**	**12 (35.3%)**	
M-GAPP score, median [IQR]	**1.0 [1.0, 2.0]**	**4.0 [2.0, 5.0]**	**<0.001**
M-GAPP score <3 (*N* = 53), n (%)	**48 (90.6%)**	**5 (9.4%)**	**<0.001**
M-GAPP score ≥3 (*N* = 19), n (%)	**9 (47.4%)**	**10 (52.6%)**	

GAPP, Grading system for Adrenal Pheochromocytoma and Paraganglioma; IQR, interquartile ranges; MD, moderately differentiated; M-GAPP, modified GAPP; PASS, Pheochromocytoma of the Adrenal Scaled Score; PD, poorly differentiated; PPGL, pheochromocytoma and paraganglioma; WD, well differentiated.

Significant results (*P* < 0.05) are in bold.

The AUC of the GAPP score for predicting metastatic PPGL (0.728) was similar to that of the PASS score (0.753) (*P* = 0.757). The AUC of the M-GAPP score (0.822) was significantly higher than that of the GAPP score (*P* = 0.012) and similar to that of the PASS score (*P* = 0.411) (Table 7).

**Table 7:** Receiver-operating characteristic analyses of three scoring systems for predicting metastatic potential in pheochromocytoma and paraganglioma.

Variable	AUC	95% CI	Improvement vs. GAPP score	*P* value	Improvement vs. PASS	*P* value
GAPP score	0.728	0.610–0.826	Ref	Ref		
PASS	0.753	0.637–0.847	0.025	0.757	Ref	Ref
M-GAPP score	**0.822**	**0.714–0.902**	**0.094**	**0.012**	0.069	0.411

AUC, area under curve from receiver-operating characteristic analysis; CI, confidence interval; GAPP, Grading system for Adrenal Pheochromocytoma and Paraganglioma; M-GAPP, modified GAPP; PASS, Pheochromocytoma of the Adrenal Scaled Score.

Significant results (*P* < 0.05) are in bold.

### MFS according to the baseline GAPP score, PASS, and M-GAPP score

**Fig 2:**
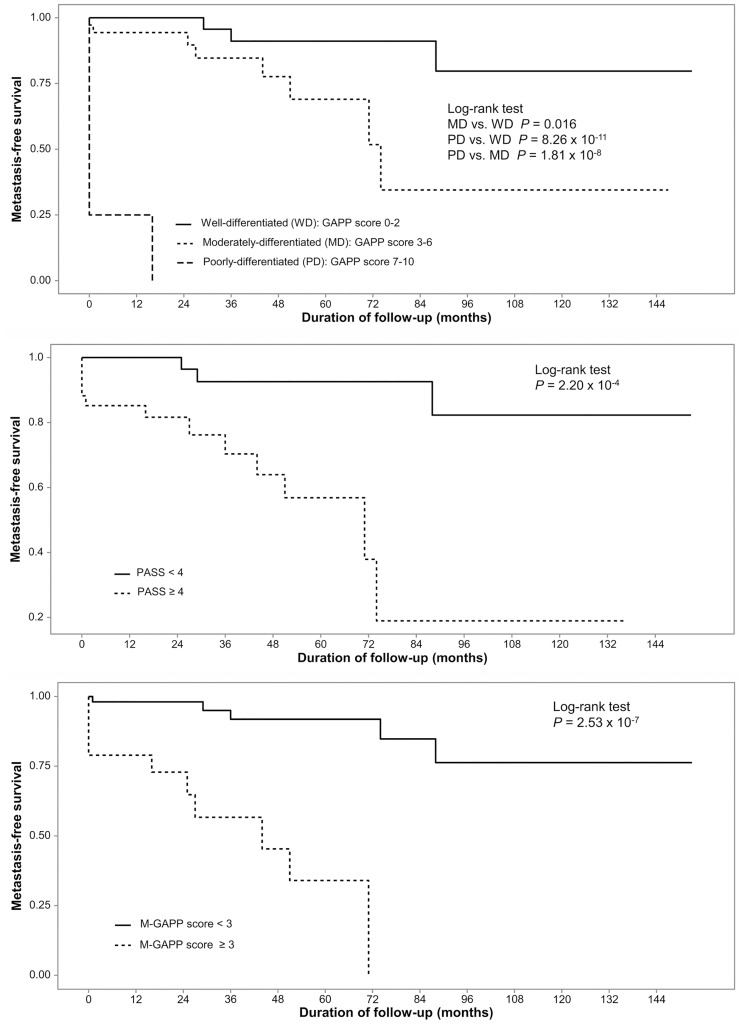
Metastasis-free survival analyses according to the Grading system for Adrenal Pheochromocytoma and Paraganglioma (GAPP) score, Pheochromocytoma of the Adrenal Scaled Score (PASS), and modified GAPP (M-GAPP) score at the initial operation.

**Fig 3:**
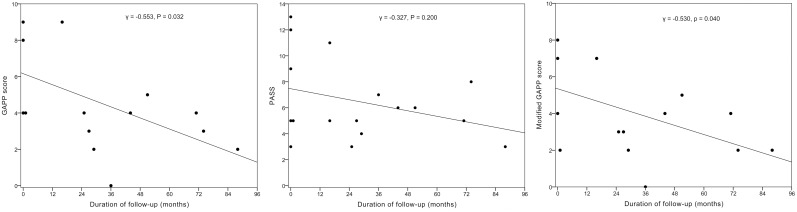
Correlation of the Grading system for Adrenal Pheochromocytoma and Paraganglioma (GAPP) score, Pheochromocytoma of the Adrenal Scaled Score (PASS), and modified GAPP (M-GAPP) score at baseline with the time (months) to metastasis after the initial operation.

All 3 scoring systems significantly predicted MFS (Fig 2). The GAPP (γ = -0.553, *P* = 0.032) and M-GAPP scores (γ = -0.530, *P* = 0.041) negatively correlated with the MFS, whereas the PASS score did not (γ = -0.327, *P* = 0.200) (Fig 3). These results suggest that all 3 systems, especially the GAPP and M-GAPP scores, may predict MFS of PPGL.

## Discussion

Our longitudinal study with a mean follow-up duration of approximately 4 years suggested that the GAPP classification might be a validated system for the prediction of metastatic potential and that our modified GAPP classification including a loss of SDHB staining might result in an improved ability to predict metastatic potential. The baseline GAPP score, PASS, and M-GAPP score were all higher in the metastatic group than in the non-metastatic group. The predictive ability of the M-GAPP score was better than that of the GAPP score, and was similar to that of the PASS. Higher GAPP and M-GAPP scores, but not PASS, were associated with a shorter MFS. Collectively, these findings suggest that, out of the 3 scoring systems, the M-GAPP might be the most useful for the prediction of metastasis in PPGL.

Determining the metastatic potential of PPGL, which is particularly important for guiding therapeutic interventions and patient management, primarily depends on histology. However, malignant PPGL can be diagnosed only after the development of metastases, which can sometimes occur as long as 20 years after the initial surgery [18]. To overcome this shortcoming, the PASS and GAPP systems were suggested to stratify primary tumors according to the risk of metastasis. Although these systems provide a reasonable prediction of metastases, several limitations still prevent either system from being generally accepted or officially endorsed [12]. In particular, the PASS had been applied only to pheochromocytoma and had a very poor concordance among expert pathologists in a validation study [10]. On the other hand, the GAPP has so far not been validated and does not include the presence of mutations in SDHB, which strongly correlates with metastatic potential [6, 11, 12].

Regarding the validation of the GAPP classification, the GAPP score was higher in the metastatic group and negatively correlated with the MFS, 90.6% of WD tumors were non-metastatic, all PD tumors were metastatic, and the MFS significantly differed between the 3 differentiation types of the GAPP classification in the present study. Therefore, the GAPP classification might be useful to predict metastases, consistent with the results of a previous study [9]. However, there were some differences between the previous and present studies. First, 9.4% in the present study and 3.6% (4 of 111) of WD PPGLs in the previous study revealed metastases. Second, a smaller proportion (22.2%) of MD PPGLs in the present study revealed metastases than in the previous study (21 of 35 MD PPGLs, 60.0%) (*P* = 0.003). Third, only 2 parameters in the present study, in contrast to all 6 GAPP parameters in the previous study, were significantly associated with metastatic potential on multivariate analysis. Fourth, the tumor capsule was mostly incomplete or absent in our cases [3], and the assessment of cellularity was not easy, with potentially high inter-observer variation, similar to for the Ki-67 labeling index. These results highlight several limitations of the GAPP classification.

Additionally, another limitation of the GAPP is that assessment of mutations in the SDHB gene is not included. A previous study showed that none of the WD PPGLs and 10 of 13 (77%) PPGLs with a loss of SDHB staining were metastatic [9], suggesting that a combination of the GAPP classification and SDHB IHC staining might be useful to predict metastases. However, due to the limited cases with negative SDHB staining among MD and PD PPGLs, the loss of SDHB staining was not included to the GAPP parameters in previous study [9, 11]. Although metastatic PPGLs showed more loss of SDHB staining than non-metastatic PPGLs in the present study, 6.3% of WD PPGLs also showed negative SDHB staining, and 6 of 11 (54.5%) PPGLs with a loss of SDHB staining were non-metastatic. These results indicate that a loss of SDHB staining *per se* is not sufficient to predict metastasis; hence, we included it as one of M-GAPP parameters. Finally, we modified the GAPP by combining some useful parameters of the original GAPP and the loss of SDHB IHC staining.

When compared with the PASS and GAPP classifications, 52.6% of PPGLs with M-GAPP ≥3 revealed metastases, while only 35.3% of PPGLs with PASS ≥4 and 30.0% of PPGLs with GAPP ≥3 revealed metastases. The M-GAPP score, but not PASS, negatively correlated with the MFS, and the predictive ability of the M-GAPP score was greater than that of the GAPP score. Most of the improvement was seen in the specificity (84.2% for M-GAPP vs. 50.9% for GAPP). Collectively, the M-GAPP system was superior in the prediction of metastatic potential of PPGLs than the other 2 scoring systems.

Our study has several limitations that should be addressed in future studies. First, PPGLs are rare neuroendocrine tumors, and we were hence only able to include a small number of Korean patients from our single medical center. Thus, further multicenter studies that include larger number of PPGLs from various ethnic groups are needed. Second, the predictive ability of the M-GAPP classification, particularly in terms of its sensitivity, should be further improved. Herein, our main aim was to validate the GAPP, so we did not consider the inclusion of other known clinical or genetic parameters reflecting metastatic potential such as age, location, size, methoxytyramine levels, and/or other molecular markers [11, 18]. Furthermore, although the loss of SDHB IHC staining can predict the SDHB mutation, it can be associated with other SDHA, SDHC, and SDHD mutations [14]. Tumors with SDHA, SDHC, or SDHD mutations revealed lesser aggressive clinical behaviors than those with SDHB mutation [19], so lack of specificity of loss of SDHB IHC staining only for SDHB mutation can be major limitation of M-GAPP classification. Thus, further comprehensive research is needed to improve the predictive scoring system of PPGLs through combinations of potential clinical-histological-genetic parameters including SDHB mutation with the M-GAPP system.

## Conclusion

Our data indicate that the GAPP classification might be a validated system for the prediction of metastatic potential. Moreover, the M-GAPP classification, which includes a loss of SDHB staining, might improve the ability to predict metastatic potential. Such risk stratification might be useful for personalized management of and as a screening strategy for PPGLs, as it could reduce both the costs of long-term follow-up and the risk of disseminated disease.

## Supporting information

S1 File Detailed information for participant-level data of this study. (XLSX)

S1 Table Pheochromocytoma of the Adrenal Scaled Score (PASS). (DOCX)

S2 Table Association of the individual parameters of the Pheochromocytoma of the Adrenal Scaled Score (PASS) at the initial operation with metastasis occurrence in pheochromocytoma and paraganglioma. (DOCX)

S3 Table Number of tumors with loss of succinate dehydrogenase gene subunit B (SDHB) immunohistochemical (IHC) staining according to the GAPP score, PASS, and M-GAPP score. (DOCX)
