# Supplementary material for: Validation of pathological grading systems for predicting metastatic potential in pheochromocytoma and paraganglioma
Source: PLoS One. 2017 Nov 8;12(11):e0187398. doi: 10.1371/journal.pone.0187398 (PMC5678867; doi:10.1371/journal.pone.0187398)
Supplement: S1 Table — (DOCX) [file pone.0187398.s002.docx]

**Supporting Information**

**S1 Table. Pheochromocytoma of the Adrenal Scaled Score (PASS)**

| PASS parameters | Score if present (no. of points assigned) |
| --- | --- |
| Large nests or diffuse growth | 2 |
| Central (middle of large nests) or confluent tumor necrosis | 2 |
| High cellularity | 2 |
| Cellular monotony | 2 |
| Tumor cell spindling (even if focal) | 2 |
| Mitotic figures >3/10 high-power fields | 2 |
| Atypical mitotic figure(s) | 2 |
| Extension into adipose tissue | 2 |
| Vascular invasion | 1 |
| Capsular invasion | 1 |
| Profound nuclear pleomorphism | 1 |
| Nuclear hyperchromasia | 1 |
| Total maximum score | 20 |
