# Supplementary material for: Validation of pathological grading systems for predicting metastatic potential in pheochromocytoma and paraganglioma
Source: PLoS One. 2017 Nov 8;12(11):e0187398. doi: 10.1371/journal.pone.0187398 (PMC5678867; doi:10.1371/journal.pone.0187398)
Supplement: S2 Table — (DOCX) [file pone.0187398.s003.docx]

**Supporting Information**

**S2 Table. Association of the individual parameters of the Pheochromocytoma of the Adrenal Scaled Score (PASS) at the initial operation with metastasis occurrence in pheochromocytoma and paraganglioma**

| PASS parameters | Univariate | | Multivariate | |
| --- | --- | --- | --- | --- |
|  | HR (95% CI) | *P* | HR (95% CI) | *P* |
| Large nests or diffuse growth | **6.37 (1.43-28.27)** | **0.015** | 3.33 (0.67-16.12) | 0.135 |
| Central (middle of large nests) or  confluent tumor necrosis | **5.07 (1.74-14.83)** | **0.003** | **3.53 (1.07-11.70)** | **0.039** |
| High cellularity | NA |  |  |  |
| Mitotic figures >3/10 HPF | **12.48 (2.28-68.32)** | **0.004** | **10.27 (1.49-70.60)** | **0.018** |
| Atypical mitotic figure(s) | NA |  |  |  |
| Profound nuclear pleomorphism | 0.61 (0.22-1.71) | 0.347 |  |  |
| Nuclear hyperchromasia | 2.93 (0.93-9.22) | 0.067 |  |  |
| Extension into adipose tissue | **5.41 (1.85-15.87)** | **0.002** |  |  |
| Vascular invasion | **3.48 (1.18-10.26)** | **0.024** | **3.47 (1.06-11.32)** | **0.040** |
| Capsular invasion | 2.07 (0.73-5.83) | 0.169 |  |  |
| Cellular monotony | 1.71 (0.54-5.44) | 0.361 |  |  |
| Tumor cell spindling (even if focal) | 1.95 (0.58-6.49) | 0.279 | **4.35 (1.07-11.70)** | **0.039** |

CI, confidence interval; HPF, high-power field; HR, hazard ratio; NA, not applicable.

Significant results (*P* < 0.05) are in bold.
