# Supplementary material for: Validation of pathological grading systems for predicting metastatic potential in pheochromocytoma and paraganglioma
Source: PLoS One. 2017 Nov 8;12(11):e0187398. doi: 10.1371/journal.pone.0187398 (PMC5678867; doi:10.1371/journal.pone.0187398)
Supplement: S3 Table — (DOCX) [file pone.0187398.s004.docx]

**Supporting Information**

**S3 Table. Number of tumors with loss of succinate dehydrogenase gene subunit B (SDHB) immunohistochemical (IHC) staining according to the M-GAPP score, PASS, and GAPP score**

| GAPP score | | | PASS | | | M-GAPP score | | |
| --- | --- | --- | --- | --- | --- | --- | --- | --- |
|  | Non-  metastatic  (*N* = 6) | Metastatic  (*N* = 5) |  | Non-  metastatic  (*N* = 6) | Metastatic  (*N* = 5) |  | Non-  metastatic  (*N* = 6) | Metastatic  (*N* = 5) |
| WD (0-2) | 2 (33.3%) | 0 (0.0%) | < 4 | 4 (66.7%) | 0 (0.0%) | < 3 | 2 (33.3%) | 0 (0.0%) |
| MD (3-6) | 4 (66.7%) | 2 (40.0%) | ≥ 4 | 2 (33.3%) | 5 (100.0%) | ≥ 3 | 4 (66.7%) | 5 (100.0%) |
| PD (7-10) | 0 (0.0%) | 3 (60.0%) |  |  |  |  |  |  |

GAPP, Grading system for Adrenal Pheochromocytoma and Paraganglioma; MD, moderately differentiated; M-GAPP, modified GAPP; PASS, Pheochromocytoma of the Adrenal Scaled Score; PD, poorly differentiated; WD, well differentiated.
